# Data Privacy Protection Based on Micro Aggregation with Dynamic Sensitive Attribute Updating

**DOI:** 10.3390/s18072307

**Published:** 2018-07-16

**Authors:** Yancheng Shi, Zhenjiang Zhang, Han-Chieh Chao, Bo Shen

**Affiliations:** 1 Key Laboratory of Communication and Information Systems, School of Electronic and Information Engineering, Beijing Municipal Commission of Education, Beijing Jiao Tong University, Beijing 100044, China; syc123321@sina.cn (Y.S.); bshen@bjtu.edu.cn (B.S.); 2 School of Information Science and Engineering, Fujian University of Technology, Fuzhou 350118, China; hcc@mail.ndhu.edu.tw; 3 College of Electrical Engineering & Computer Science, National Ilan University, Yilan City, Yilan County 260, Taiwan; 4 Department of Electrical Engineering, National Dong Hwa University, Hualien 97401, Taiwan

**Keywords:** micro aggregation, privacy protection, dynamic update, sensitive attributes

## Abstract

With the rapid development of information technology, large-scale personal data, including those collected by sensors or IoT devices, is stored in the cloud or data centers. In some cases, the owners of the cloud or data centers need to publish the data. Therefore, how to make the best use of the data in the risk of personal information leakage has become a popular research topic. The most common method of data privacy protection is the data anonymization, which has two main problems: (1) The availability of information after clustering will be reduced, and it cannot be flexibly adjusted. (2) Most methods are static. When the data is released multiple times, it will cause personal privacy leakage. To solve the problems, this article has two contributions. The first one is to propose a new method based on micro-aggregation to complete the process of clustering. In this way, the data availability and the privacy protection can be adjusted flexibly by considering the concepts of distance and information entropy. The second contribution of this article is to propose a dynamic update mechanism that guarantees that the individual privacy is not compromised after the data has been subjected to multiple releases, and minimizes the loss of information. At the end of the article, the algorithm is simulated with real data sets. The availability and advantages of the method are demonstrated by calculating the time, the average information loss and the number of forged data.

## 1. Introduction

As the Internet and Big Data have been rapidly developing ample concern focusing on protecting personal privacy, which has become one of the most popular research areas, is on the rise. For the purposes of medical services, scientific research, business strategies, information sharing, trend predictions, et cetera, many organizations that own huge amounts of personal data have needs to publish or share the data [1]. If the original personal data was not processed in any way, it must be cracked or leaked [2,3]. The main direction of contemporary data security research is how to process data in ways that both protect personal privacy along with meeting the needs described above. Mainstream methods of processing data is data generalization [4,5,6] that aims to aggregate data and generalize an accurate value to an interval within a group of data. By doing this, a potential hacker cannot deduct the accurate personal sensitive data to achieve privacy protection. The key of such methods, of generalization, is the development of aggregation (clustering) [7,8] rules, which should be designed in the way that guarantees the usability while protecting client privacy.

The following example will demonstrate the above process. In medical research, some hospitals would make diagnosis records (left part of Table 1) publicly available to the related scientific research organizations. In this example, it is assumed that every piece of individual data shown is presented in the form of a tuple (one row of the table) and has a fixed structure: including Name, Age, Zip Code, and Disease Type. In terms of the attribute Name, as it is an identity attribute (unique identification of the entity) the publicized data will exclude such sensitive attributes for privacy’s sake. The attributes of Age and Zip Code are classified as quasi identity attribute [9] (indirect identification of the entity) and the attribute of Disease Type is classified as sensitive attribute. If only the identity attribute were deleted the risks of leak would still exist. For instance, a hacker knowing an individual’s age and zip code may be able to conclude that a specific individual (identifiable by name) has dyspepsia. This could be triangulated from the data table without the attribute of Name (i.e., Bob). A method dividing data, into many groups, and generalizing the quasi identity attribute within each group (right part of Table 1), the hacker would have difficulty connecting the disease Bob has, even knowing Bob’s age and zip code [10,11]. Consider the following situation: Bob and Alice both have dyspepsia and if a hacker cannot be sure which data entry is Bob’s they could still deduct that Bob has the disease. From the above example, it is clear that the key of the generalization is to design the rules to get the groups assuring the security of the personal sensitive data (diversifying data sensitive attribute within a group) and maximize the usability of the data (maximizing the similarity of elements in the group). Currently, mature generalization algorithms are K-anonymity, L-diversity, and L-diversity P-sensitive. All of them are static methods, which could allow information leaks under the circumstances of data updating and multiple publication. For instance, if data was published twice and only one piece of data was added the second time. If hacker knew who was added by chance, he/she must be able to obtain the personal sensitive data by the differences between the two publications. In addition to the static feature, all of those methods determine the rules of aggregation on fixed parameters, which cause unnecessary information loss. In terms of dynamic generalization methods—for instance, M-invariance—forged data is added to assure the unification of multiple publication. This may also cause information loss.

This paper presents a privacy protection method to support dynamic update based on micro aggregation [12,13,14]. This method uses the concept of distance between tuple and information entropy increase of a group together as the input. In order to find a best cluster that not only the availability of information is guaranteed, but also the privacy protection is realized [15,16]. The method also proposes a dynamic update scheme to realize privacy protection when the data is changed. A Laplace noise mechanism is also applied to protect the sensitive attributes of result set. The rest of this paper is organized as follows: Section 2 presents the related work of data anonymity privacy protection. Section 3 proposes a clustering algorithm based on the micro aggregation and lists some relevant concepts. The pseudo code of the algorithm is given. Section 4 illustrates the details of the dynamic update method and the implementations of noise mechanism. The pseudo code of the algorithm is also given. Section 5 selects the real world data set and do the simulation. The experimental results have been verified by calculation time, average information loss, and the number of forged tuples. Finally, conclusions and future work are presented in Section 6.

## 2. Related Work

In recent years, academic researchers have done a lot of studies on privacy protection. In the privacy protection literature, there are three fields of work with seemingly different goals. The first field is about privacy by policy. The second field is about privacy by statistics [17]. The third field is about privacy by cryptography. This paper and the literatures contained is mainly focus on the second field. All the research is carried out under a core idea—maximizing the availability of the data with the condition of individual privacy not being leaked. The basic theory of privacy protection in the second field is k-anonymity [18,19]. After anonymity processing, the method requires that each record cannot be distinguished from the other k-1 records in a data table, but the k-anonymity method does not take into account protecting the sensitive attributes. The result can be attacked by an attacker who has background knowledge. L-diversity [20,21] theory is based on the k-anonymity considering the protection of sensitive attributes, which ensures that each group contains L different sensitive attribute values. Attackers can only infer the sensitive information of the target by the probability of 1/L. A reference proposed a method to protect data privacy by clustering in static environment, which decides the equivalent groups in the data set by clustering analysis and finally achieves privacy protection [22]. Imagining another situation, L-diversity based on the k-anonymity deals with the sensitive attributes, and makes sure that there are L attributes in an equivalent group at least, but if the L attributes almost concentrating on some certain attributes, consequently, attackers can infer the result of the personal privacy in high probability, so the theory which is called L-diversity P-sensitive [23,24] comes into being. The main idea of the theory is the l-diversity p-sensitive algorithm not only guarantees each QI group has L sensitive attributes, but also makes sure the biggest probability of any sensitive attributes in each equivalent group is less than P which is defined at the beginning of the method [25]. Although the literatures mentioned above had made great contribution on privacy protection, those methods only suit for static data, in other words, they did not consider the need for multiple releases of the dynamic data.

In the field of data dynamic updating [26,27], the data set is constantly decreased or increased with different needs. Here are some effective methods of data dynamic updating which can achieve privacy protection no matter how many versions the result are released. Byun and Sohn firstly proposed a method [28] of dynamic updating that prevents privacy being leaked when data set is re-released. Kui puts forward an m-invariance [29,30,31] generalization principle dealing with the situations of insertion or deletion of dynamic data. Zhang presents a NC m-invariance [32] algorithm to solve the problem of dynamic numerical sensitive attributes based on the theory of M–invariance, which can transform the dynamic numerical sensitive attributes to classified sensitive attributes. Wang [33] introduces a method which can keep sensitive attributes consistent in the different versions caused by continuous re-release and proved its efficient utility. In reference [34], an anonymous method using the concepts of data decomposition is proposed. The core idea of this method is separating sensitive attributes and quasi-identifier attributes into two parts, and associated by the parameter G_ID.

## 3. Clustering Model Based on Micro Aggregation

This section proposes a clustering algorithm based on micro aggregation [35,36]. It solves the problem of choosing suitable equivalent groups with flexible balance between personal privacy and data availability. At the same time, this method considers the distance value between tuples and the concept of information entropy together as a criterion in the process of clustering. It is different from the L-diversity or P-sensitive theories mentioned above. This algorithm does not choose the parameter L or P as a criterion for clustering which may cause some unnecessary information loss. At the end of the section, the pseudo code is given and the rationality of the algorithm is analyzed.

### 3.1. The Measurement of Tuples ‘Attribute Distance’

This section discusses how to define the distance between two tuples. The result will be used as input for clustering in the next section. At first, the definition of distance between two tuples is presented below: let *t*_1_, *t*_2_ ∊ T (T is the entire data set), the distance between tuples *t*_1_ and *t*_2_ is the sum of all quasi-identifier attributes’ distance. For example, the distance between bob and Alice in Table 1 is the sum of distances in age and zipcode.

(1) $$\mathrm{dis}\left(t1,t2\right)=\sum_{i=1}^{QI}d\left(t_{1i},t_{2i}\right)$$

The next problem is how to define the distance between two quasi identifier attributes form different tuples [37]. In this article, quasi identifier attributes are divided into two main categories, the continuous attributes and the discrete attributes. The discrete attributes are divided into ordinal attributes and nominal attributes. For the distance of the continuous attribute, the difference of value range will cause a great impact on the result when calculating the distance value between two tuples. To suppress the different value range between attributes, it is necessary to standardize the value of continuous attributes into [0, 1]. The formula for conversion is as follow. *x*_max_ and *x*_min_ are the maximum and minimum value of the attribute *x*, *x_i_* is the attribute value of the tuple, *x_d_* is the final value after normalization. The distance between tuple1 and tuple2 is defined below:

(2) $$x_{d}=\frac{x_{i}-x_{\min}}{x_{\max}-x_{\min}}$$

In order to calculate the distance between the discrete attributes, the value of the discrete attribute must be numeric. The discrete attributes are divided into ordinal attributes and nominal attributes. If there is a certain difference or size relationship between two values, the attribute is called ordinal attribute:

(3) $$d\;(t_{1i},t_{2i})=|x_{d1}-x_{d2}|$$

For example, when a tuple has an evaluation attribute which contains several certain options like excellent, good, moderate, and poor. So the evaluation attribute is ordinal attribute. To quantify the value of ordered attributes, we use the rank value of ordered attributes [38]. Refer to the distance measurement given by Ferrer [39], we improve the distance measurement and list as below. The weights of the attribute rank is define as *W*_1_′2, *W*_2_′3, …, *W_n_*_−1_′*n* (*W_i_*_−1_′*i* >0; *i* = 1, 2, 3, …, *n*). The weight indicates the degree of dissimilarity between the attribute values. For the different values a and *b*(*a* ≤ *b*) from an ordinal attribute *A_i_*, their distance is defined as:

(4) $${\mathrm{dis}}_{\mathrm{Ai}}\left(a,b\right)=\frac{\sum_{j=a+1}^{b}W_{j-1\prime j}}{\sum_{j=2}^{\left|D\left(A_{i}\right)\right|}W_{j-1\prime j}}$$

where *D* (*A_i_*) is the value range of *A_i_*. Another category of discrete attributes is nominal attributes. If there is neither a differential relationship nor a size relation between the values of a property, it is called a nominal attribute. For example, when a tuple has the religion attribute which contains Buddhism, Catholicism, Islam and so on. There are no differences between relationship and size. Therefore, the religious attribute is a nominal attribute. In this paper, we use the method of numerical transformation proposed in [40] to preprocess the nominal attributes. An *n* × *n* square matrix (Table 2) is used to represent the processed nominal attribute with *n* different values:

(5) $$\mathrm{dis}\left(X,Y\right)={\left(S_{1x}-S_{1y}\right)}^{2}+{\left(S_{2x}-S_{2y}\right)}^{2}+{\left(S_{3x}-S_{3y}\right)}^{2}$$

After the conversion, the distance of nominal attribute between two tuples *X* and *Y* is defined as above. When the nominal attribute has n values, the above polynomial will have n items. After those analyses, the final distance between two tuples will be calculated, and the distance value will be the input for the micro aggregation in following steps deciding which tuple belongs to an equivalent group.

### 3.2. The Measurement of Sensitive Attribute Entropy

The above definitions of the distance between tuples make sure that the most similar tuples will join an equivalent group so the information loss is minimal. However, the sensitive attributes are not protected. The basic way in other micro aggregation algorithms is considering clustering at first, and then considering the protection of sensitive properties. For example, when an equivalent group is generated based on the distance between tuples, some other tuples which is not belong to this group must be readjust into the equivalent group in order to achieve some certain standards like L-diversity or P-sensitive for the purpose of protecting sensitive attributes. Therefore, this will cause information loss inevitably. This article uses information entropy as one of the indicators of clustering for micro aggregation, deciding the clustering result together with the definition of distance from tuples. The following definition of information entropy is listed below. Here will also discuss the rationality of the algorithm:

(6) $$H\left(G\right)=E[-\log p_{i}]=-\sum_{i=1}^{n}p_{i}\log p_{i}$$

As we all know, from the information point of view, if, the value of the above formula is 0 when there is no uncertainty. When the probability is distributed evenly, the value reaches the maximum. This just meets the requirements of sensitive attribute protection, if the sensitive attribute values from an equivalent group are concentrated in a certain value, then the entropy value of the equivalent group is small. On the contrary, if the sensitive attribute values from an equivalent group are distributed on average, the entropy value of the equivalent group reaches the maximum.

Suppose that the equivalent group *G* of the data table contains n different sensitive attribute values *S*_1_, *S*_2_, …, *S_n_*, *P_i_* represents the probability of a sensitive attribute value *S_i_*. The *H* (*G*) is the entropy value of the equivalent group *G*, *H* (*G*′) is the entropy value of the equivalent group *G*′. *G*′ just adds another one tuple than the equivalent group *G*. The definition *E* (*G*, *G*′) represents the entropy value increase from the equivalent group *G* to *G*′. This paper uses the *E* (*G*, *G*′) as a parameter of the clustering to choose which tuple is the best one (the biggest entropy increase) to join the equivalent group in order to protect individual privacy.

Consider the following situations, any equivalent group with an entropy of 0 must lead to the disclosure of privacy, because there is no uncertain information for the attacker who wants to deduce the personal privacy in the result set. On the contrary, the biggest entropy increase makes sure that this tuple is the best one to join the equivalent group starting for the purpose of maintaining the best privacy protection in the result:

(7) $$E\left(G,G^{\prime }\right)=H\left(G\right)-H\left(G^{\prime }\right)$$

### 3.3. Micro Aggregation Clustering Algorithm Description

This section uses the idea of the micro aggregation algorithm [41] and let the distance between tuples, the entropy increase in the equivalent group together as inputs for the clustering algorithm. Moreover, two parameters are involved in the process of clustering deciding either the information availability or individual privacy is more important to the data receiver. The core idea of the algorithm is to find a series of equivalent groups using the inputs mentioned above. Each group starting with just a tuple, and continuously select the most appropriate record (not only similar to the group but also reach the aim to protect individual sensitive attributes) from the overall data to join the group. The pseudo code of this algorithm is also listed below.

The most important thing in the clustering process is to find a numerical value deciding which tuple is the best choice to join the equivalent group. This section defines a function as below:

(8) $$C\left(G,G^{\prime }\right)=A_{p}\cdot E\left(G,G^{\prime }\right)-B_{u}\cdot dis\left(G_{\mathrm{centroid}},t\right)---E\left(G,G^{\prime }\right)\neq 0$$

*G* represents the original equivalent group, when the clustering algorithm begin, the equivalent group just selects a random tuple from the data table. The tuple t is another new tuple in the data set. The purpose of this algorithm is to find a tuple t to join the equivalent group *G* and thus a new equivalent group *G*′ is formed. *E*(*G*,*G*′) represents the entropy increase from the original equivalent group *G* to the new group *G*′. *G*′ just contains the new tuple t. *E*(*G*,*G*′) not equal to 0 ensures the equivalent group *G* can’t always contain the same sensitive attribute in the beginning of clustering. *G*c represents the average value of the equivalent group *G*, dis (Gc,t) represents the distance between the new tuple t and the average value of the equivalent group *G*.

The parameter *A*_p_ and *B*_u_ are used to flexibly determine either the information availability or individual privacy is more important to the data receiver. By selecting different values for this two parameters, the orientation of the result will be changed. Consider the following situation, if just thinking about the distance concept at first, and then let the L-diversity and P-sensitive as mandatory parameters for the result separately. This is very likely to cause information loss in the equivalent group.

The algorithm in this chapter is still based on the *K* anonymity theory and an equivalent group contains *K* tuples at least. It is necessary to select the parameter *K* at the beginning of the clustering to determine the minimum size of each equivalent group. We use a set *Q* to represent the equivalent groups after clustering. The pseudo code of the algorithm is given below:
**Algorithm 1**Inputs: Original data table T, quasi-identifier attributes, k-anonymous parameter K.Output:The equivalent groups after generalization, anonymous data table.BEGINIF(the number of tuples in T <= K) ReturnLet Q = ∅ // Empty sets of equivalent groupsWhile(the number of tuples in T>k)** Randomly select a tuple t from T, T=T−{t},G={t}**** While(the number of tuples in G<k)****$\;\;\;t^{\prime }=\mathrm{Arg}\_\max\;C(G,G\cup {\{t}^{\prime }\})t^{\prime }\in T$****   Gk=Arg_max C(G,G∪Gk)Gk∈Q****$\;\;\;\mathrm{If}(C(G,G\cup {\{t}^{\prime }\})>C(G,G\cup Gk))$****$\;\;\;\;\mathrm{Than}\;T={T-\{t}^{\prime }\},G=G\cup \{t^{\prime }\}$****  Else Q=Q−Gk,G=G∪Gk**** End While**** Q=Q∪G**End WhileWhile(there still remain tuples in T)** Randomly select a tuple t from T, T=T−{t}**** Gk=Arg_max C(G,G∪Gk)Gk∈Q****$\;Q=Q-G,G=G\cup \{t^{\prime }\}$**** Q=Q∪G**End While**Each tuple’s quasi-identifier values are replaced by the centroid of this group in Q**END

This paragraph provides the necessary explanation for the algorithm above. Step 2 defines an empty set *Q* which will save equivalent groups. The set *Q* will be the result after clustering. In step 3, *T* indicates the total data. When the number of tuple in *T* is more than *k*, the loop continues. Step 4 shows that a t is randomly taken from *T* as an equivalent group *G* and remove *t* from *T*. Steps 5–10 are the key processes in the micro aggregation algorithm. Step 5 shows that as long as the number of t in *G* is less than *k*, loop will be continued. Step 6 aims to find the most suitable tuple t to join the equivalent group *G* based on the value of Equation (8) presented in last paragraph. Step 7 aims to find the most suitable equivalent group *G_k_* which contained in the set *Q*. Step 8 compares either the step 6 or step 7 is the best way to do with the equivalent group *G* (let *t* join to *G* or let *G* join to *G_k_*). In step 9, *t* will be removed form *T* and join to the equivalent group *G* when the condition is established. In step 10, *G_k_* will be removed form *Q* and fused with *G* when the condition is established. In step 12, *G* will be added to the set *Q*. Steps 14–19 illustrate when there is only a few tuples (less than *k*) in *T*, choosing a tuple *t* randomly and forming a new equivalent group *G*. Let the group *G* compare to each existing group in set *Q* for the purpose of finding the most suitable equivalent group in *Q* to join. Step 20 makes the quasi-identifier attributes of each groups in set *Q* all replaced by the centroid that will finally complete the anonymization of the data.

## 4. Dynamic Update Based on Micro Aggregation

For classical K-anonymity and L-diversity, they are only suitable for static data but invalid for dynamic environment. Although the m-Invariance theory can dynamically update the data like adding or deleting tuples, the process will iterate all the tuples when adding, deleting or modifying. It will cause a great redundancy. Besides, if the data has been released for several times, there are more and more forged data in each equivalent group. This will reduce the availability of information. This section proposes a dynamic scheme based on the micro aggregation algorithm mentioned above to achieve the dynamic update like adding, deleting a tuple t in a more effective and flexible way. This chapter mainly involves the following points: Dynamic adjustment after micro-aggregation clustering, forged data, Laplace noise scheme.

### 4.1. Dynamic Adjustment after Micro-Aggregation Clustering

Based on the micro-aggregation algorithm mentioned in the previous section, the steps 14–18 illustrate how the remaining tuples in *T* to choose which equivalent group *G_k_* is the best choose to join. If we just simply let the new tuples (adding to *T*) use the previous way to cluster, the equivalent groups in set *Q* will be more and lager as the adding tuples increased.

As the equivalent groups become more and more huge, the information loss will increase simultaneously in a group. No matter what attribute’s value the new tuple has, it will always join to the existing groups and finally reach the limit of one group. This chapter puts forward a method of dynamic clustering based on the previous micro-aggregation algorithm. When clustering process has been completed and a new tuple needs to update, the beginning of the new process is same with the algorithm (steps 14–18) in the last section but creates another collection table *W*. A cache of the new tuple will saved in the table *W*. The pseudo code of the algorithm is given below:

Here provides the necessary explanations for the algorithm above. Step 4 uses table *W* to save the cache of a new tuple. Step 5 defines a callback function, and uses the new tuple as the input to the micro aggregation algorithm we proposed in last section. After the function, the set *Q_w_* which contains the equivalent groups from the cache table *W* is formed (if the number of tuples belong to table *W* less than *K*, this process will not execute). In steps 6 and 7, it find out the most suitable equivalent group *G_k_* which belongs to *Q*. And the equivalent group *G_w_* that belongs to *Q_w_*. In step 8–step 13, if the new tuple is more suitable to join the equivalent group *G_k_* than the group *G_w_* , it will let the new tuple and the *G_k_* combine together as a expansion group which belongs to the set *Q*. On the contrary, if the new tuple is more suitable to join the equivalent group *G_w_* than the group *G_k_*, the tuples involved need to be deleted from the set *Q*, and the new tuple becomes a member in the *G_w_*. After that, *G_w_* will be transformed from *Q_w_* to *Q*. The following examples explain the process above.
**Algorithm 2**Inputs: Clustered data table T, New tuple t, Call back function(x) Algorithm 1.Outputs: The updated data table T.BEGIN**1. Let W = ∅ **2. While(there is a new tuple needs to update)3. Select the new tuple t,G={t}4. Let t→W // The set W save the cache of t5. Callback function→input:table W,output set Qw6. Gk=Arg_max C(Gk,G∪Gk)Gk∈Q7. Gw=Arg_max C(Gw,G∪Gw)Gw∈Qw8. If(C(Gk,Gk∪G)>C(Gw,Gw∪G))9.   Q=Q−Gk,Gk=Gk∪{t}10.  Q=Q∪Gk11. Else 12.  Drop((t∈Gw)⊂Q),Qw=Qw−Gw13.  Q=Q∪Gw14. End WhileEND

Table 3 (left part) is the original equivalent group based on the data table, and Table 3 (right part) is the cache table Tw that saves the new tuples adding to the data set. The GID 1 in the cache table Tw represents some tuples that joined into the table earlier (those tuple also saved in *T*). When a new tuple t1 (age 25 zip 27,000 disease flu) needs to be added, it will decides which group is most suitable for the new tuple. In the current situation depicted in Table 4, the new tuple join the original equivalent group 2, and also make a cache in the table *W*. When another new tuple t2 (age 26 zip 29,000 disease headache) needs to be added, it will examine whether there has any suitable group in cache table *W*. we supposed that *G_w_* is a equivalent group in *Q_w_*. When a new tuple join to the cache table *W*, it find out the new tuple *t* join to group *G_w_* is better than join to *G_k_* and the new tuple will added to the *G_w_*. After that, the *G_w_* will be removed from *Q_w_*, and becomes a member in *Q*.

As shown in Table 5, the tuple t1 and t2 are combined into a new equivalent group, and drop them from the set *Q* and the set *Q_w_* respectively. The new group will be added to the set *Q* at last.

### 4.2. Dynamic Protection of Sensitive Attributes

The consistency of sensitive attribute’s signature from each equivalence group ensures that the privacy of each group cannot be divulged. The definition of signature as below: If *G* is an equivalent group from generalization table *T*, the signature of *G* is set of each equivalent group’s sensitive attribute values (the signature of G_ID 3 from Table 5 is {flu, headache}). The signature of each group must be same in different versions. For example, the signature of G_ID 3 from Table 5 is {flu, headache}. When a new tuple needs to be added to this group, the signature of the group may become {flu, headache, dyspepsia}. If an attacker knows who will join the group in advance, he will infer the sensitive attribute value of the person is dyspepsia by comparing the differences between two versions. After the dynamic clustering mentioned above, the same problem must be solved.

When a new tuple join to a group, the signature changes will expose personal privacy information [42]. The core idea of anonymous privacy protection is to ensure that the personal sensitive information cannot be distinguishable from other tuples. If the attacker can’t distinguish two tuples, the basic way of privacy protection is realized [43]. In this paragraph, a real-time monitoring process that detecting the changes from each group is defined. It will creating a forged tuple into a group while some condition is satisfied. The description of this process as follows:
**Algorithm 3**Inputs: Clustered data table T, the status of the equivalent group.Outputs: The updated data table T with forged tuple.BEGIN1.While(Group change⇒t→St∈S)2. If(t_F⊆G)$3.\;\;t\_F\to {t\_F}^{'}(S\_\mathrm{random}\in S\neq St)$4. Else 5.  G∪G{t_F→S_random∈S≠St}6.  Q=Q∪GwEnd WhileEND

In Step 1, the process detects the changes in each group. When there is any change likes adding a new tuple into a group or deleting a tuple from a group or modifying an existing tuple, it will check out if there has any forged data in this group. Situation 1: the equivalent group already has the forged tuple. Let the sensitive attribute value of the forged tuple t change to other sensitive attribute value Sr randomly. Situation 2: there is no forged tuple in the group. Step 5 creates a forged tuple t with the random sensitive attribute value Sr adding to the group. The same process is also applied to the delete operation. When deleting a tuple from any equivalent group, there are also two situations as above. As for the modifying, it will be replaced by the deleting and adding steps.

In addition, this article uses two tables to store quasi identifier attributes and sensitive attributes, respectively. For example, quasi identifier attributes are stored in Table 6 (left part) and sensitive attributes are stored in Table 6 (right part). The GID will connect this two tables. This method weakens the connection between quasi identifiers and sensitive attributes and still maintains the trend or availability of the data. The process of clustering, dynamic updating, are all implement by the data owner. After those processes, the data owner will publish the data like Table 6. The sensitive attribute’s percentage of each group is generated according to the distribution from the original group. The receiver can use this data to mine, predict, or analyze some related problems.

Compare to the m-invariance theory, the biggest advantage in this method is there just only one forged data in each equivalent group. As the tuples continue to increase or we choose a big anonymous parameter number k, this method will cause less impact on the availability of information. In terms of privacy protection, there just makes two tuples are indistinguishable by adding the forged data. In other words, an attacker has 50% chance to infer the personal privacy information. In order to solve this problem, this article makes use of the Laplace noise mechanism which will be introduced in next section.

### 4.3. The Laplace Noise Mechanism

In this paper, all the processes like clustering, updating, data saving or some other mentioned above are all carried out by the data owner. For example, the data owner maybe a data system of the hospital or wearable equipment’s manufacturer [44,45]. When some authorized institutions need to obtain the data saved in the data owner for the purpose of outsourcing calculation, data mining, analyzing or forecasting, they will sent a request to the data owner to get the anonymous data. The data owner will check the authority of the receiver at first, and add the Laplace noise to the sensitive attribute percentage value from each equivalent group in the anonymous data. The availability of data will not be greatly reduced by adding the noise, because the noise added to each sensitive attribute distribution’s percentage value from each group with different parameter based on some rules. In the previous section, the strength of privacy protection is just making two tuples indistinguishable when the data table is updating. The method presented in this chapter will greatly improve this situation.

The Cytoplasm distribution is selected as the source of noise. Cytoplasm noise is a random value that satisfies the Cytoplasm distribution. In the domain of differential privacy, the Cytoplasm noise mechanism is a common way to protect personal privacy. As an innovation, a method using Cytoplasm noise to protect privacy based on dynamic micro aggregation updating is proposed in this paper. The probability density function of the distribution is expressed as follows:

(9) $$f(x|\mu ,b)=\frac{1}{2b}e^{\frac{\left|x-\mu \right|}{b}}$$

The probability accumulative function of distribution as follows can be deduced by the probability density function:

(10) $$F(x|\mu ,b)=\left\{ \begin{array}{l} 0.5\times e^{-\frac{\mu -x}{b}},x<\mu \\ 1-0.5\times e^{\frac{x-\mu }{b}},x\geq \mu \end{array}\right.$$

The parameters *μ* and *b* represent the expected value and variance of the distribution. The parameter *μ* generally selects 0 as its value because the result should be floated based on the original value. The parameter b represents the degree of privacy protection based on the sensitive attribute distribution value. Since the range of the cumulative probability function is [0, 1], so before Laplace noise is generated, the random values of a uniform distribution should be first generated in the interval [0, 1]. By solving the inverse function of the cumulative probability function, the noise that meet the distribution of the Laplace can be generated. Let the random variable ε meets the uniform distribution in the interval [0, 1], the inverse accumulation distribution function can be deduced as follow:

(11) $$x=\left\{ \begin{array}{l} b\ln(2\varepsilon )+\mu ,\varepsilon <0.5\\ \mu -b\ln(2(1-\varepsilon )),\varepsilon \geq 0.5 \end{array}\right.$$

The above formula is a piece wise function, in the noise generation stage it will result in an increase of a certain degree of complexity. So let the random variable *ε* meets the uniform distribution in the interval [−0.5, 0.5], and the inverse accumulation distribution function can become a single formula as below:

(12) x=μ−b×sign(ε)×ln(1−2abs(ε))

(13) $$N=N_{\max}-N_{\mathrm{current}}$$

When the random number ε in the interval [−0.5, 0.5] is generated, the variable x as the input for the probability density function can be obtained based on the above formula. Let the variable *x* as the input for the probability density function, and then the cytoplasm noise value *n* is generated. Because the expectation of the noise is 0, so it will cause the probability density function reaching its maximum value. In order to let the noise float by the distribution value of the original sensitive attribute, the value of the noise *N* is defined as Equation (13). The levels of noise should be different because of different distribution values of sensitive attributes, so here defines the weight of each noise that based on the original probability distribution of sensitive attributes. It makes sure that different distributions of sensitive attribute has the different noise value. *N_i_*′ is the final noise value that add to the each sensitive attribute’s distribution, and the *W_i_* is each noise’s weight calculated by the original distribution value of sensitive attributes:

(14) $$N_{i}^{\prime }=W_{i}\times N_{i}W_{i}=\;\frac{D_{i}}{\sum_{i=1}^{n}D_{i}}$$

Consider the following situation, if an attacker ask the data owner for the anonymous data frequently, he or she can infer the real result value of sensitive attribute’s distribution through calculating the mean value of the result. The reason is each noise that added to the final result obeys the Laplace distribution with an expected value. If the data owner just let the authorized recipients request for the result without restricting, they will simply calculate the real result and the Laplace noise mechanism loses it’s meaning of protection, so as the data owner like hospital’s information database, manufacturer of wearable devices, some third-party cloud computing platforms and so on, they should restrict each receiver’s query times during some time based on each receiver’s authorization level.

## 5. Experiment and Result Analysis

This article uses the data set Adult in the UCI Machine Learning Repository, it consists of U.S. Census data. Some records with missing values in the data set are removed. After that, it has 45,222 records and contains 14 attributes. This experiment selects eight properties: age, gender, race, status marital, number education, country native, class work and occupation. Here selects occupation as a sensitive attribute. As for The Laplace noise mechanism, we use the Apache Commons implementation “Laplace Distribution” and the noise is generated by extracting a random sample from the distribution. This section defines a concept for calculating the information loss after clustering and the result will be analyzed and compared to other algorithm. This section also analyzes the availability of the algorithm and compares the execution time based on different experimental parameters. The hardware environment of the experiment was an Intel(R) Core(TM) i7-4710MQ CPU @ 2.50 GHz (Lenovo, Beijing, China) equipped with 12 GB RAM and running the Win 10 OS. 

At the beginning of this section, the execution time of the algorithm will be discussed. In order to display the effect of the algorithm intuitively, we only choose the number of 5000, 10,000 and 20,000 tuples separately as the input for the Micro aggregation algorithm as comparison. The clustering run time of each condition is shown in the diagram below.

**Figure 1:**
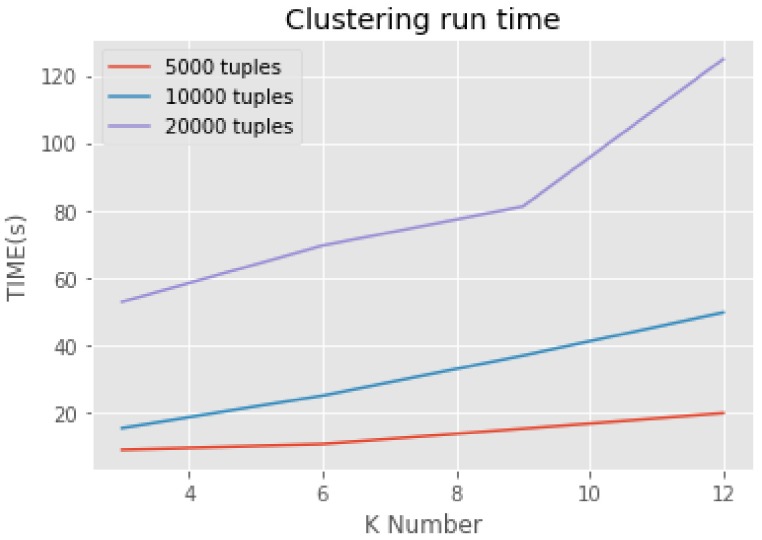
Clustering run time.

Here we let the parameters A_p_ and B_u_ as 0.6, 0.4. These two parameters determine whether the degree of privacy protection or the data availability is more important. No matter under what circumstances, the execution time of clustering will increase as we choosing a bigger anonymous parameter K. When choosing a large data set, the execution time of the algorithm grows rapidly as K increases (Figure 1). Compared with ordinary clustering algorithm, the execution time of the algorithm has increased to an extent within an acceptable range. The reduction of information loss will compensate for the increase of execution time. The next paragraph measures the information loss from the following aspects.

This paragraph only discusses information loss in static way, in other words, the experimental results of micro aggregation in Section 3.3. The final set *Q* contains the equivalent groups. Each tuple *t* has the value *C* (*G*, *t*) for the purpose of finding the most suitable tuple *t*′ to join the equivalent group *G*. *C* (*G*, *t*) contains two factors: the distance value between the tuple t and the center of equivalent group *G* and the entropy increase which decided by the sensitive attribute in each group. The information loss is caused by the generalization when clustering, so it just takes the distance value between the tuple t and the equivalent group into consideration:

$$L_{AVG}=\frac{\sum_{i=1}^{Q.size}\sum_{j=1}^{G.lenth}t_{dis}}{\left|T\right|}$$

**Figure 2:**
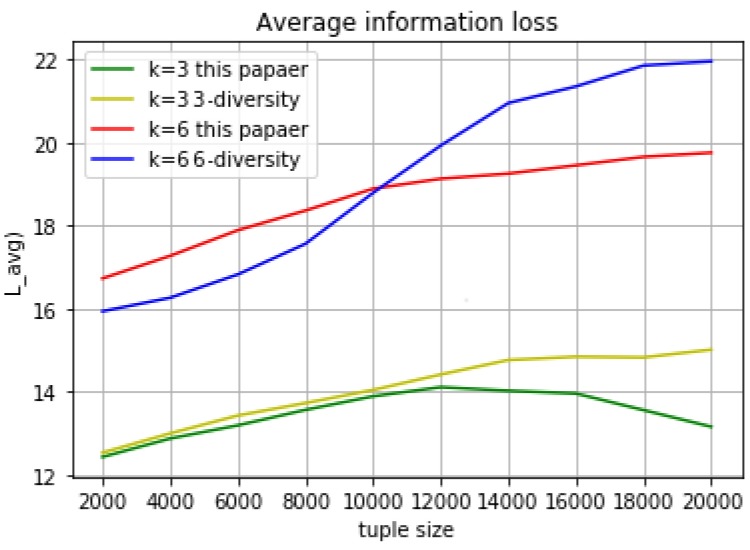
Average information loss.

Each tuple has a distance value when it join to an equivalent group. The average information loss in a group is calculated as: the sum of distance value from a group divides the total number of the group’s tuples. The total average information loss is calculated as: the sum of each equivalent group’s average information loss divides total number of tuple in the data table. The experimental results are shown above (Figure 2). Compared with k- anonymous l-diversity (in the same set of experiments, we use the same value for K and L, because the range of sensitive attributes is much larger than the number of tuples in the group), the average loss of information value will significantly increase when we choose the bigger anonymous parameter K in both algorithm, but the peak value will reach stability faster as the tuple size growing in this paper’s algorithm.

**Figure 3:**
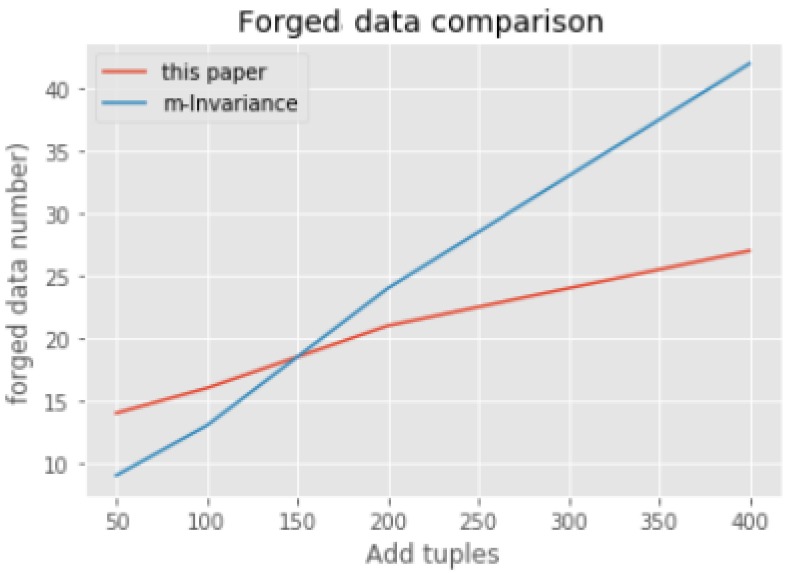
Forged data comparison.

In addition, here we verify the experimental results by the number of forged data from the perspective of dynamic update in Section 4. We compare the quantity of forged data between m-invariance and our dynamic update algorithm. We selects the same 5000 records for clustering. After that, we choose different size of tuples to join the result above as comparison based on the dynamic update method (those incremental records are processed one by one according to the method in Section 4.1 and 4.2) in this paper and m-invariance. The chart as below (Figure 3). When there just a little new tuples adding to the result of clustering, the number of the forged tuple of this paper’s method are bigger than the m-invariance algorithm. With the growth of added tuples, the forged tuples in our dynamic update algorithm are significantly less than m-invariance algorithm. Because there just only one forged tuple in each equivalent group at most, no matter how many new tuples are added to the result of clustering. With the growth of the added data, the effect of the dynamic update algorithm are better than the m-invariance algorithm.

## 6. Conclusions and Future Work

Different needs based on health care, scientific research, business plan, data sharing, trend prediction, policy making, the data owners, such as government, enterprises, and equipment manufacturers, may need to publish the data that they have. For some reasons, they need to update the data and release the different versions of the result. If the original data is released without processing, it will lead to personal information being exploited by others.

This paper studies the data privacy protection problem and proposes a dynamic update algorithm based on micro aggregation. This method uses the concepts of distance between tuple and information entropy increase of a group together as the input. In order to find a best cluster that not only the availability of information is guaranteed, but also the privacy protection is realized. The method also proposes a dynamic update scheme to realize privacy protection when the data is changed. A Laplace noise mechanism is also applied to protect the sensitive attributes of result set. In addition, this article saves the quasi identifier attribute and sensitive properties separately and gives the different result to the receiver based on their limits of authority. In a word, this algorithm ensures the information availability after data anonymity and efficiently reduce information loss with dynamic update function.

In the future work, we will continue to learn these following aspects: (1) How to minimize the risk of privacy leakage in different versions of the data; (2) The distance between tuple is the key to the whole algorithm, so how to optimize the distance property is a major research in the next; (3) Based on the different privileges level for receivers, how to introduce differential privacy protection mechanism to query result set is also needs to be considered.

## Figures and Tables

**Table 1:** Diagnosis records and anonymous generalization result.

Name	Age	Zip	Disease	GID	Age	Zip	Disease
Bob	21	12,000	dyspepsia	1	(21,22)	12–14 k	dyspepsia
Alice	22	14,000	bronchitis	1	(21,22)	12–14 k	bronchitis
Andy	24	18,000	flu	2	(23,24)	18–25 k	flu
David	23	25,000	gastritis	2	(23,24)	18–25 k	gastritis
Gary	41	20,000	flu	3	(36,41)	20–27 k	flu
Helen	36	27,000	gastritis	3	(36,41)	20–27 k	gastritis

**Table 2:** Nominal attributes matrix.

Religion	*S*_1_	*S*_2_	*S*_3_
Buddhism	$\sqrt{0.5}$	0	0
Catholicism	0	$\sqrt{0.5}$	0
Islam	0	0	$\sqrt{0.5}$

**Table 3:** The original equivalent group and the cache table W.

GID	Age	Zip	Disease	Cache Table *W*			
1	(21,22)	12–14 k	dyspepsia	GID	Age	Zip	Disease
1	(21,22)	12–14 k	bronchitis	1	x1	xx000	dyspepsia
2	(23,24)	18–25 k	flu	1	x2	xx000	bronchitis
2	(23,24)	18–25 k	gastritis	…	…	…	…

**Table 4:** The equivalent group and the cache table W (when t1 is added).

GID	Age	Zip	Disease	Cache Table *W*			
1	(21,22)	12–14 k	dyspepsia	**GID**	**Age**	**Zip**	**Disease**
1	(21,22)	12–14 k	bronchitis	1	x1	xx000	dyspepsia
2	(23,25)	18–27 k	flu	1	x2	xx000	bronchitis
2	(23,25)	18–27 k	gastritis	1	25	27,000	flu
2	(23,25)	18–27 k	flu	…	…	…	…

**Table 5:** The equivalent group and the cache table W (when t2 is added).

GID	Age	Zip	Disease	Cache Table W			
1	(21,22)	12–14 k	dyspepsia	**GID**	**Age**	**Zip**	**Disease**
1	(21,22)	12–14 k	bronchitis	1	x1	xx000	dyspepsia
2	(23,24)	18–25 k	flu	1	x2	xx000	bronchitis
2	(23,24)	18–25 k	gastritis	2	25	27000	flu
3	(25,26)	27–29 k	flu	2	26	29000	headache
3	(25,26)	27–29 k	headache	…	…	…	…

**Table 6:** Quasi identifier attributes and the result set.

GID	Age	Zip	GID	Disease	Percentage
1	(21,22)	12–14 k	1	dyspepsia	a%
1	(21,22)	12–14 k	1	bronchitis	b%
2	(23,24)	18–25 k	2	flu	c%
2	(23,24)	18–25 k	…	…	…
